# Altered alpha/beta desynchronization during item–context binding contributes to the associative deficit in older age

**DOI:** 10.1093/cercor/bhac219

**Published:** 2022-06-25

**Authors:** Anna E Karlsson, Myriam C Sander

**Affiliations:** Max Planck Institute for Human Development, Center for Lifespan Psychology, Berlin, Germany; Department of Psychology, Humboldt-Universität zu Berlin, Berlin, Germany; Max Planck Institute for Human Development, Center for Lifespan Psychology, Berlin, Germany

**Keywords:** aging, alpha, associative memory, oscillations, theta

## Abstract

It is proposed that older adults have difficulties to bind item and context and to recruit deep, elaborative processing during encoding. Senescent changes in the oscillatory foundations of these processes are currently unclear. We recorded electroencephalography during item–context memory formation in younger (*n* = 57) and older (*n* = 55) adults. At test, we assessed memory for the items and the item–context pairs and examined encoding-related activity based on how much information was recovered at retrieval (miss < item–only < pair). Item memory was comparable between age groups while pair memory was reduced in the older adults. Theta synchronization and alpha/beta desynchronization increased linearly with the amount of information available. Single-trial theta power could not predict subsequent item memory, but predicted pair memory in an age-invariant manner, in line with a mechanism supporting associative memory. In contrast, single-trial alpha/beta power predicted both item and pair memory, in line with a mechanism reflecting the depth of information processing, and predicted pair memory less well in the older than the younger adults. Thus, theta and alpha/beta oscillations contribute differently in shaping the contents of memories and reduced processing capacity contributes to episodic memory decline in older age.

## Introduction

Episodic memory is the ability to construct, maintain, and recover memories of past events in great detail, specifying where and when the event took place (Tulving 2002). Memory impairments are common complaints in older age (Newson and Kemps 2006), and episodic memory is particularly affected by senescent cognitive decline (Nyberg et al. 2012). A long-standing hypothesis in the literature proposes that age-related declines in episodic memory are due to impairments in the binding of distinct pieces of information during memory formation (Chalfonte and Johnson 1996; Naveh-Benjamin 2000), which is essential for episodic memory. Experimental work has shown that older adults often perform equally well as their younger counterparts in item-recognition tasks. At the same time, age differences are greater for various sorts of associative information, such as item–item and item–context associations (e.g. Naveh-Benjamin 2000; Howard et al. 2006; Bender et al. 2010; for reviews, see Spencer and Raz 1995; Old and Naveh-Benjamin 2008; Koen and Yonelinas 2014; Nyberg and Pudas 2019). However, despite accumulating evidence for an associative deficit in older age, it remains elusive whether senescent changes in encoding-related processes can account for these observations (see also Craik and Rose 2012; Sander et al. 2021).

Successful memory formation has been extensively studied with the subsequent memory effect (SME) paradigm, where neural activity during encoding of trials that are later remembered are contrasted against trials that are later not remembered, with the aim to isolate neural activity associated with successful memory formation (Paller and Wagner 2002). Previous work utilizing the SME approach on electroencephalography (EEG) data in younger adults has provided convincing evidence for the contribution of oscillatory activity to subsequent memory performance (for reviews, see Nyhus and Curran 2010; Hanslmayr et al. 2016). In particular, successful memory formation has been associated with power increases (i.e. synchronization) in the theta frequency range (~3–7 Hz) and power decreases (i.e. desynchronization) in the alpha/beta (~8–30 Hz) frequency range.

Synchronization in the theta band has been shown to support successful episodic memory formation (e.g. Staudigl and Hanslmayr 2013; Backus et al. 2016; Sander et al. 2020) and retrieval (e.g. Guderian and Düzel 2005; Gruber et al. 2008; Herweg et al. 2016). In addition, the medial temporal lobe (MTL), a region heavily involved in associative memory (O’Reilly and McClelland 1994; Simons and Spiers 2003; Moscovitch et al. 2016), has been identified as the source region of SME in the theta band (Hanslmayr et al. 2011). Theoretical accounts of these and similar findings propose that theta supports item–context binding via the coordination of neural communication within the hippocampus and between the hippocampus and cortical regions (Clouter et al. 2017; Wang et al. 2018; for reviews, see Fell and Axmacher 2011; Lisman and Jensen 2013). Thus, theta synchronization during encoding may provide an index of binding strength. However, others have reported power decreases (i.e. negative SME) rather than power increases in the theta band (e.g. Michelmann et al. 2018). As pointed out recently (Herweg et al. 2020), positive theta SME has been observed in most studies explicitly assessing associative memory, but whether theta synchronization plays a special role in associative memory formation remains unclear. Therefore, the first objective of this study was to assess whether theta synchronization supports associative memory formation in particular, as compared to simple item memory formation.

Alpha/beta desynchronization has been associated with the level of elaborative processing during learning (Hanslmayr et al. 2009, 2016; Hanslmayr and Staudigl 2014), and is parametrically modulated by the amount of information available at encoding and retrieval (e.g. Griffiths et al. 2019; Karlsson et al. 2020; Martín-Buro et al. 2020; see also Griffiths et al. 2021). Accumulating evidence suggests that alpha/beta desynchronization regulates the flow of information through task-relevant cortical assemblies, promoting depth of information processing and ultimately determines the amount information available at encoding and retrieval (Hanslmayr et al. 2012; Parish et al. 2018). Accordingly, previous work has reported that alpha/beta desynchronization at retrieval is stronger for associative memory than for item recognition (Martín-Buro et al. 2020; see also Karlsson et al. 2020). However, to the best of our knowledge, it remains to be tested whether the same pattern holds during encoding. Thus, a second objective of this study was to assess whether alpha/beta desynchronization during encoding scales with the amount of information available at retrieval (i.e. item recognition < associative memory; cf. Karlsson et al. 2020).

The great majority of studies investigating SME in older adults have used functional resonance imaging (fMRI). In line with an associative deficit, altered encoding-related activity in the hippocampus and the surrounding MTL have been reported in older adults (e.g. Daselaar et al. 2006; Dennis et al. 2008). In particular, reduced SMEs were observed in the hippocampus in the older compared to the younger adults during the formation of associations (Dennis et al. 2008). At the same time, no age differences in hippocampal SME were found for simple item memory formation. In addition, altered functional connectivity between the hippocampus/MTL and cortical regions was observed in the older adults (Daselaar et al. 2006; Dennis et al. 2008). Together, these and similar findings suggest that older adults’ difficulties to form associations during learning is linked to altered activity in and between regions involved in associative memory formation. However, others have reported age-invariant SME, or even greater SME in older adults, in the hippocampus and other cortical regions implicated in episodic memory (e.g. de Chastelaine et al. 2016; Zheng et al. 2018; for review, see Maillet and Rajah 2014). However, the discrepancies between studies may potentially be due to differences in how well the task could separate familiarity-based item recognition from recollection (Jacoby 1991; Yonelinas 1994), as suggested when directly contrasting item and pair memory formation (Dennis et al. 2008).

Similarly, the very few EEG studies available investigating age differences in SME also report inconclusive results (Sander et al. 2020; Strunk and Duarte 2019; for review, see Werkle-Bergner et al. 2006). In an item-recognition task, theta SMEs were not found in younger or older adults and marginally reduced alpha/beta SMEs were reported in the older compared to the younger adults (Strunk and Duarte 2019). However, theta synchronization should be especially involved in associative memory formation, and thus less modulated by item recognition. Similarly, age differences should be more pronounced for associative memory as compared to item recognition (Old and Naveh-Benjamin 2008; Craik and Rose 2012). Nonetheless, in a study assessing associative memory using a cued recall task, no age group differences were found in theta or alpha/beta SME either, despite reliable age differences in associative memory performance (Sander et al. 2020). However, the authors demonstrated that alpha/beta power and the cortical thickness of the inferior frontal gyrus (IFG) jointly predicted single-trial memory success. The IFG has previously been identified as a source region of the alpha/beta SME (Hanslmayr et al. 2011; see also Hanslmayr et al. 2014) and is strongly implicated in episodic memory formation and semantic elaboration in particular (for reviews, see Paller and Wagner 2002; Kim 2011). Importantly, Sander and colleges (2020) reported that the majority of participants showing reduced alpha/beta desynchronization accompanied with reduced IFG thickness were indeed older adults. Thus, contrary to the associative deficit hypothesis, theta synchronization appears not to contribute to age differences in episodic memory formation. However, attenuated alpha/beta desynchronization and reduced IFG thickness is related to associative memory decline in older age. These findings resonate nicely with the levels-of-processing framework (Craik and Lockhart 1972; Craik and Rose 2012), proposing that, on a cognitive level, older adults have problems initiating elaborative, deep processing of incoming information due to a reduction in neural resources. Unfortunately, the few studies available make it difficult to draw strong conclusions and the discrepant findings may depend on how well the task was able to assess associative memory.

**Fig. 1:**
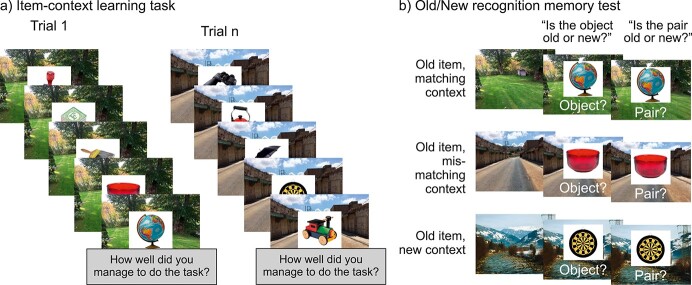
Experimental paradigm. a) During learning, objects were presented superimposed on pictures of outdoor scenes and participants were instructed to imagine using the object in the place depicted. b) In a subsequent surprise recognition memory test, old and new objects were presented on old (matching/mismatching) or new scenes. Participants first judged whether they had seen the object before and then whether they had seen the specific object–scene pair before. Matching scenes corresponded to the scene on which the object was presented during encoding. Mismatching scenes corresponded to old scenes from encoding that were previously paired with another object. The mismatch condition emphasized the need to base the pair memory judgment on the association between the item and the context, and not simply on a feeling of familiarity for the constituent parts of the pair (adapted from Karlsson et al. 2021).

To summarize, previous work suggests that older adults have difficulties binding item and context information and recruiting efficient information processing necessary for deep elaboration during learning, but observations are inconclusive. Surprisingly, despite 2 promising candidate mechanisms, i.e. theta synchronization and alpha/beta desynchronization, indexing associative binding and depth of information processing respectively, only a few studies have investigated age differences in these mechanisms during episodic memory formation. Given this large gap in the literature, the third and main objective of this study was to investigate age differences in theta synchronization and alpha/beta desynchronization during successful formation of associative as compared to simple item memory. Directly contrasting encoding-related oscillatory signatures predictive of subsequent item recognition and associative memory in an age-comparative setting allowed us to assess how these mechanisms interact with age during the formation of memories that differ in quality at retrieval (i.e. item vs. pair memory). To this end, younger and older adults engaged in an item–context association task while being measured with EEG (see Fig. 1). They were provided with an encoding strategy that aimed to foster the binding of item and context in both age groups. At test, we assessed their memory for both the individual items and for the item–context associations. Thus, we could separate trials not remembered at all (i.e. misses) from trials for which only the item was later remembered, without memory for the associated context (i.e. item–only memory), and trials for which the item–context association was remembered (i.e. pair memory).

We first isolated the frequency ranges where neural activity was predictive of subsequent memory performance on the group level (misses < item – only memory < pair memory). Next, we followed up on the group-level pattern by examining how within-person, single-trial power fluctuations in these frequency ranges predicts what can later be retrieved using logistic mixed effects modeling. The single-trial analysis allowed us to further scrutinize the pattern observed on the group level and examine whether theta synchronization and alpha/beta desynchronization support item–only and pair memory differently while assessing the effect of age. Given the proposed role of theta synchronization for associative memory formation, we predicted that, on the group level, theta synchronization at encoding would increase linearly with the amount of associative information available at retrieval (misses < item – only memory < pair memory). However, if theta synchronization indexes associative binding, single-trial theta power should predict subsequent pair memory, but not item memory. Furthermore, in line with the associative deficit in older age, we expected single-trial theta power to predict pair memory less well in the older than in the younger adults. Considering alpha/beta desynchronization as a proposed index of the depth of information processing, we predicted that, on the group level, alpha/beta desynchronization would increase linearly as a function of the amount of information available at retrieval (misses < item – only memory < pair memory). Since these power modulations should be invariant to the associative nature of that information, we expected single-trial alpha/beta power to mirror the group-level analysis and to predict both subsequent item–only and pair memory. However, if older adults have difficulties to engage deep, elaborative processing, which presumably affects pair memory formation the most, we expected single-trial alpha/beta power to predict item memory equally well across age groups, but pair memory less well in the older compared to the younger adults.

## Methods

We have reported aspects of these data in a previous study assessing theta–gamma coupling during associative memory formation (Karlsson et al. 2021). Methods and analyses that are relevant for the current study are repeated here. The samples used in the 2 studies are identical.

### Participants

Younger (*n* = 65) and older (*n* = 72) healthy, right-handed, German-speaking adults were recruited via a database of the Max Planck Institute for Human Development to participate in the study. The provided written informed consent and was given a compensation of €12/h. A total of 25 participants were excluded due to technical problems (6), drop out (3), noisy EEG data (1), memory performance below chance (2; see below), or due to too few trials per condition (13; see “EEG recording and preprocessing”). The remaining sample consisted of 57 younger (33 females, *M*_age_ = 25.0, SD_age_ = 3.1, range 20–31 years) and 55 older adults (24 females, *M*_age_ = 69.6 SD_age_ = 3.6, range 64–76 years). To further characterize the sample, the participants completed a demographic questionnaire and additional cognitive tests. The Digit Symbol substitution test (Wechsler 1955) was used to measure processing speed, and younger adults attained a significantly higher score (*M* = 66.23; SD = 10.88) than older adults (*M* = 49.91; SD = 10.14; *W* = 4,336, *z* = 6.49, *α* = 0.05, *P* < 0.001). The Spot-a-Word test (Lehrl et al. 1995) was used to measure verbal knowledge, and older (*M* = 28.95; SD = 3.21) showed significantly better performance than younger (*M* = 22.42; SD = 3.41; *W* = 1,915, *z* = −7.61, *α* = 0.05, *P* < 0.001) adults. Finally, older adults (*M* = 28.78, SD = 1.34, range = 24–30) were assessed using the Mini Mental State Examination (Folstein et al. 1975) measuring cognitive impairment. A score of 27 or higher indicates normal cognition, whereas a score of 19–23 indicates mild cognitive impairment (O’Bryant et al. 2008). Performance in these tasks was similar to other cognitive neuroscience studies previously run at our research center (e.g. Sander et al. 2011; Fandakova et al. 2014; Karlsson et al. 2020) and showed a typical pattern of age differences. The ethics committee of the Deutsche Gesellschaft für Psychologie (DGPs) approved the study.

### Stimuli

The experiment was programmed in MATLAB (version 2016b; MathWorks Inc., Natick, MA, USA), using the Psychophysics Toolbox (Brainard 1997). The stimuli pool consisted of 498 colored pictures (400 × 400 pixels) of everyday objects (e.g. ball, teapot) and 210 colored pictures (1,280 × 960 pixels) of outdoor scenes (e.g. forest, beach).

### Experimental paradigm

The experiment consisted of 4 parts, a prelearning phase, a learning phase, a postlearning phase, and a retrieval phase, completed during 1 day with short breaks in between the sessions, including a 40-min lunch break after the learning phase. For the purpose of the present analysis, we focus on the learning and retrieval phases. However, note that the objects and scenes presented in the learning phase, and thus serving as “old” in the retrieval phase, were presented individually in a target detection task during the prelearning phase performed in the MRI scanner. In the target detection task, participants passively viewed all stimuli, presented individually for 2 s, and indicated with a button press whenever a white fixation cross presented on top of the stimuli turned red. Furthermore, the objects were also presented in the postlearning phase in a similar target detection task.

The learning phase (see Fig. 1 for illustration of the paradigm) was performed in a dimly lit room that was electromagnetically and acoustically shielded. Participants’ neural activity was recorded with EEG and their eye movements measured with an eye-tracker. Prior to the task, the participants received instructions and completed 2 short practice rounds that could be repeated if necessary. In addition, each experimental session started with written instructions on the screen. The experimenter initiated the session with a button press. During encoding, 250 randomly drawn objects and 50 randomly drawn scenes were presented together in an item–context association task. Each trial started with a jittered fixation cross (~1–1.5 s), followed by the presentation of a scene. Next, 5 objects were presented (á 3 s) sequentially superimposed on the center of the same scene, and each object was separated by a fixation cross (for 2 s). To ensure explicit attempts to associate objects and scenes and promote binding in both age groups (e.g. Craik and Rose 2012; Bastin et al. 2013), participants were instructed to imagine using the presented object in the place depicted in the scene. After the presentation of the 5 objects, participants indicated on a 3-point scale how well they had managed to do the imagery task.

At test, participants were presented with the old objects intermixed with 150 randomly drawn new objects, presented on either a new (*n* = 100) or an old scene (*n* = 50). This resulted in 5 test conditions: old objects presented on their original scene from study (match context condition, *n* = 100 trials), old objects on old but mismatching scenes from study (mismatch context condition, *n* = 100 trials), old objects on new scenes (old-new context condition, *n* = 50 trials), new objects on old scenes from study (new-old context condition, *n* = 100 trials) and new objects on new scenes (new-new context condition, *n* = 50 trials). Each test trial started with a fixation cross (0.5 s) followed by the presentation of a scene (1 s). Next, an object was presented superimposed on the center of the scene and participants were first required to respond if the object was old or new (max 3 s) and subsequently if the specific object-scene pair was old or new (max 4 s). Importantly, the match/mismatch conditions made the retrieval of the “association” between item and context crucial, since reliance on a feeling of familiarity for the constituent parts could not discriminate between old and new item–context pairs. In addition, conjointly assessing item and pair memory allowed us to separate trials for which only the item was later remembered without memory for the association (i.e. item–only memory) from trials for which the item–context association was remembered (i.e. pair memory). Responses were recorded using a response box with color-coded buttons and the mapping of color to response (“old”/“new”) was counterbalanced across participants. Finally, we constrained the occurrence of old objects and scenes in the recognition test in the following ways:

1. Of the 5 objects belonging to the same learning sequence (i.e. presented on the same scene), 2 objects were assigned to the match context condition, 2 objects to the mismatch context condition, and 1 object to the old-new context condition. Since the position of the object within the learning sequence could influence memory performance, we made sure that each condition contained an equal number of objects from each encoding position.
2. The order of trials was randomized, with the constraint that no more than 3 consecutive trials could stem from the same condition.
3. Each scene from encoding was presented 5 times (within the different test conditions, see above), but never in consecutive trials during test.

### Behavioral analysis

To first assess item chance-level performance, the proportion of correct item responses was calculated across all three context conditions (match, mismatch, old-new) with the chance level set to 0.31 (derived from the multiplication of the response probability by the proportion of trials with an old item, i.e. 0.5 × 250/400 = 0.31). Participants with performance below this chance level were excluded from the analysis. Behavioral responses were then categorized as misses (i.e. “new item” responses to old items), item–only memory (i.e. item hits followed by an incorrect pair response) and pair memory (i.e. item hits followed by a correct pair response). Note that only trials from the match and mismatch conditions were included in the analysis. To assess age differences in memory performance, response rates were analyzed in a mixed effects ANOVA, with age (younger/older) as between- and memory level (miss/item–only/pair) as within-subjects factor. Significant interactions were followed up by pairwise comparisons. The analysis was performed with R 3.5.2 (R Development Core Team 2018).

### E‌EG recording and preprocessing

The EEG was recorded with BrainVision Recorder (Brain Vision Products GmbH, Gilching, Germany) from a 61 Ag/Ag-Cl electrode-embedded cap that was positioned according to the 10–10 system (1,000 Hz sampling rate; right mastoid reference). One electrode above the forehead (AFz) served as ground. To measure eye movements for the electro-oculogram (EOG), electrodes were placed below the left eye and at the left and right outer canthi. Electrodes’ impedances were kept below 5 kΩ during the EEG preparation. In addition, participants heart rate was recorded via electrocardiogram (ECG) to remove possible cardiovascular artifacts from the EEG signal (see below).

The EEG preprocessing was performed using FieldTrip (Oostenveld et al. 2011), EEGLAB (Delorme and Makeig 2004), and custom-written MATLAB code. Before preprocessing, eye tracking and EEG data were merged along the time vectors to ensure equal time-point zero across modalities. The EEG data were filtered (4th filter order) with a passband of 1–150 Hz and rereferenced to the linked mastoid channels. The ECG data were filtered with a band-stop filter (48–52 Hz) and appended with the EEG data. Next, the data were segmented into epochs of 2 s and each epoch was visually inspected. Epochs containing strong artifacts not related to eye movements or blinks were temporarily excluded for a following independent component analysis (ICA). In addition, any channel that was strongly contaminated by artifacts was excluded. Blink, eye-movement, muscle, and heartbeat artifacts were detected using ICA (Bell and Sejnowski 1995) and removed from the signal. In addition, saccade-related transient spike potentials were identified using the COSTRAP algorithm and removed from the signal as independent components (Hassler et al. 2011). Artifact-contaminated channels and trials (determined across all epochs) were automatically identified (i) using the FASTER algorithm (Nolan et al. 2010) and (ii) by detecting outliers exceeding four standard deviations of the kurtosis of the distribution of power values in each epoch within low- (0.2–2 Hz) or high-frequency (30–100 Hz) bands, respectively. Channels labeled as artifact-contaminated were interpolated using spherical splines (Perrin et al. 1989). Next, the data were segmented into 7-s epochs, ranging from 2 s before to 5 s after stimulus onset and sorted into individual trials. A second visual inspection of each trial per participant was performed and any remaining artifact-contaminated trials were excluded from further analysis, resulting in an average of 5.95% of trials rejected. The prepocessed EEG data were subjected to time–frequency decomposition of each trial (50 ms sliding window) using a Morlet wavelet approach (wavelet width = 7 cycles), estimating spectral power from 2 to 30 Hz in steps of 2 Hz, as implemented with FieldTrip.

Finally, the data were sorted into 3 conditions of interest: misses (i.e. old objects incorrectly endorsed as new), item–only memory (i.e. “old” item responses followed by “incorrect” pair responses) and pair memory (i.e. “old” item responses followed by “correct” pair response). Thus, item–only memory reflects successful object memory without memory for the object-scene association, whereas pair memory reflects successful memory for the object-scene association. Again, note that only trials belonging to the match and mismatch conditions were included in the analyses. Participants with fewer than 10 trials per condition were excluded from further analysis, resulting in an average of 40 (min = 10, max = 82) miss trials in the younger and 38 (min = 11, max = 95) in the older adults, 54 (min = 22, max = 79) item–only trials in the younger and 70 (min = 43, max = 92) in the older adults, and 92 (min 59, max 151) pair trials in the younger and 78 (min = 48, max = 110) in the older adults.

### Subsequent memory effects on the group level

To quantify the modulation of oscillatory power during memory formation, activation data (0–3 s) were first contrasted against a prestimulus baseline period (−700 to −200 ms) within each participant. To wash out any potential prestimulus effects, baseline data were averaged over all trials. Next, on the person level, a *t*-value reflecting the difference in power between baseline and activation data was computed for each channel–frequency–time point and per each memory outcome condition (misses, item–only memory, pair memory) using single sample *t*-statistics. This step was done to normalize each individual’s power spectrum. We interpret these *t*-values as baseline-corrected power in the following. Next, to isolate encoding-related theta and alpha/beta power modulations predictive of subsequent memory outcome, the person-specific *t*-values were used to predict memory outcome (miss, item–only, pair-memory) in a univariate 2-sided, dependent samples regression analysis, where a positive effect would indicate a linear increase in power and a negative effect a linear decrease in power across memory outcomes. Initially, clusters were formed based on the resulting regression coefficient *t*-statistics for each electrode, frequency (2–30 Hz), and time point (0–3 s) and corrected for multiple comparisons via nonparametric, cluster-based, random permutation test (FieldTrip toolbox; Maris and Oostenveld 2007) on the group level. The threshold for data points to be included in a cluster was set to *P* < 0.01 and the spatial constraint was set to a minimum of two neighboring channels. Next, the significance of the cluster-level statistic (i.e. the summed *t*-values) was assessed by comparison to a permutation null distribution, which was obtained by randomly switching the condition labels and recomputing the *t*-test 5,000 times. The final cluster *P*-value (i.e. the Monte Carlo significance probability) is the proportion of random partitions in which the cluster-level statistics were exceeded. The cluster-level significance threshold was set to *P*-values below 0.025 (2-sided significance threshold).

### Single-trial power modulations on the within-person level

In a next step, we aimed to further scrutinize the linear effect identified on the group level and assess the relevance of theta and alpha/beta power modulations for item–only and pair memory success, respectively. To this end, the within-person relationship between encoding-related theta and alpha/beta power and subsequent item–only and pair memory was assessed on the single-trial level. A mixed-effects logistic regression (i.e. generalized linear mixed effects model, GLMM; Quené and van den Bergh 2008) was fit to predict single-trial memory outcomes (misses, item–only memory, pair memory) with single-trial theta and alpha/beta power (see Sander et al. 2020 for a similar approach). Single-trial power was first normalized against baseline by contrasting the baseline data, constructed as described above, against the power spectrum of each individual trial using the single sample *t* formula with a slight modification: the sample mean was replaced by single trial power (see equation 1).

(1) \begin{equation*} t=\frac{\mathrm{pow}-M}{\sqrt{S2/n}} \end{equation*}

Thus, $\mathrm{pow}$ is the single-trial power spectrum (peristimulus), $M$ is the mean power spectrum of the baseline (prestimulus) over trials, $S2$ is the variance of the power spectrum (peristimulus) over all trials, and $n$ is the number of trials. While taking the variance over trials into account, this procedure allowed us to place each individual data point in relation to the comparison distribution (i.e. the baseline data). Thus, the resulting *t*-values reflect the difference in power from pre- to peri-stimulus onset for each trial.

Next, the single-trial *t*-values were averaged over a frequency-and-region of interest (FROI), that is, channels, frequencies, and time points for which significant power differences were evident in the group-level analysis as described above. To foreshadow the group-level results, a positive (~2–6 Hz; ~0–2 s) and a negative (~8–25 Hz; ~0.2–3 s) cluster was identified (see Fig. 3b). Data averaged within the FROI defined by the “positive” cluster reflect single trial modulations relative to baseline within the theta band. We restricted the analysis to the theta range demonstrating a more sustained effect (i.e. 2–4 Hz; see Fig. 3b). Data averaged within the FROI defined by the “negative” cluster reflect single-trial modulations relative to baseline within the alpha/beta band. Both clusters showed widespread topographical distributions that varied somewhat across time points, with some channels showing a sustained effect across all time points (positive cluster: F1, Fz, FC1; negative cluster: PO7, PO3, O1, PO4, PO8, O2).

Finally, to facilitate the interpretation of the parameter estimates, the averaged *t*-values were *z*-scored across trials and centered around the mean within each participant. The *z*-scored *t*-values were then entered in the mixed-effects model predicting single-trial memory performance with single-trial theta and alpha/beta power while assessing the effect of age (see equation 2). To consider individual differences in trial numbers, between-subject differences were included as random effects. To assess the effect of age on the relationship between encoding-related activity and subsequent retrieval outcome, age was included as a fixed effect and were allowed to interact with theta and alpha/beta power. Thus, age was entered as a factor explaining the between-person variance, whereby the model assesses whether age modulates the relationship between single-trial power and subsequent memory performance. Two separate models were used to predict item–only memory (miss vs. item–only correct; item–only model) and pair memory (item–only correct vs. pair correct; pair model) respectively.

(2) \begin{equation*} \mathrm{Retrieval}\, \mathrm{outcome}\!\sim\! \mathrm{theta}\, \mathrm{x}\ \mathrm{age}+\mathrm{alpha}\, \mathrm{x}\, \mathrm{age}+\left(1|\mathrm{subject}\right) \end{equation*}

We used maximum likelihood with an Adaptive Gauss-Hermite Quadrature (nAGQ = 10) for parameter estimation (see Table 1), implemented in the lme4 package (Bates et al. 2015) in R 3.5.2 (R Development Core Team 2018). To validate the model fit over a more parsimonious model as well as the predictive value of encoding-related neural activity for subsequent retrieval outcome, we contrasted the full models with (i) constant-only models and (ii) identical models without EEG predictors (i.e. theta and alpha/beta power) by means of likelihood ratio tests.

**Table 1:** Parameter estimates for the mixed-effects model including EEG, age group, and context condition as predictors of single-trial retrieval outcome.

	Item–only memory model				Pair memory model			
	EST	STD	*Z*	PR(>|*Z*|)	EST	STD	*Z*	PR(>|*Z*|)
(Intercept)	**0.38**	**0.08**	**4.81**	**<0.001**	**0.54**	**0.04**	**12.10**	**<0.001**
Theta	0.05	0.03	1.72	0.09	**0.06**	**0.02**	**2.50**	**0.01**
Age = OA	**0.32**	**0.11**	**2.81**	**0.005**	**−0.43**	**0.06**	**−6.84**	**<0.001**
Alpha	**−0.29**	**0.03**	**−9.79**	**<0.001**	**−0.36**	**0.02**	**−15.35**	**<0.001**
Theta:Age = OA	0.06	0.04	1.50	0.14	−0.01	0.03	−0.32	0.75
Alpha:Age = OA	0.01	0.04	0.21	0.83	**0.30**	**0.03**	**9.19**	**<0.001**

*Notes*: EST = estimate, STD = standard error, *Z* = *z*-value, OA = older adults. Significant effects (*P* < 0.05) are printed in boldface. Note that OA are provided as the reference category to interpret the age effect. For example, the alpha-by-age interaction indicates that the alpha/beta power is higher (i.e. positive *z*-value) in the older adults than you would expect from the main effect of alpha/beta collapsed across age groups.

### Code availability

Data and custom written MATLAB and R code of the main analyses are available on https://osf.io/tcdkn/.

## Results

### Older adults show decreased pair memory performance

**Fig. 2:**
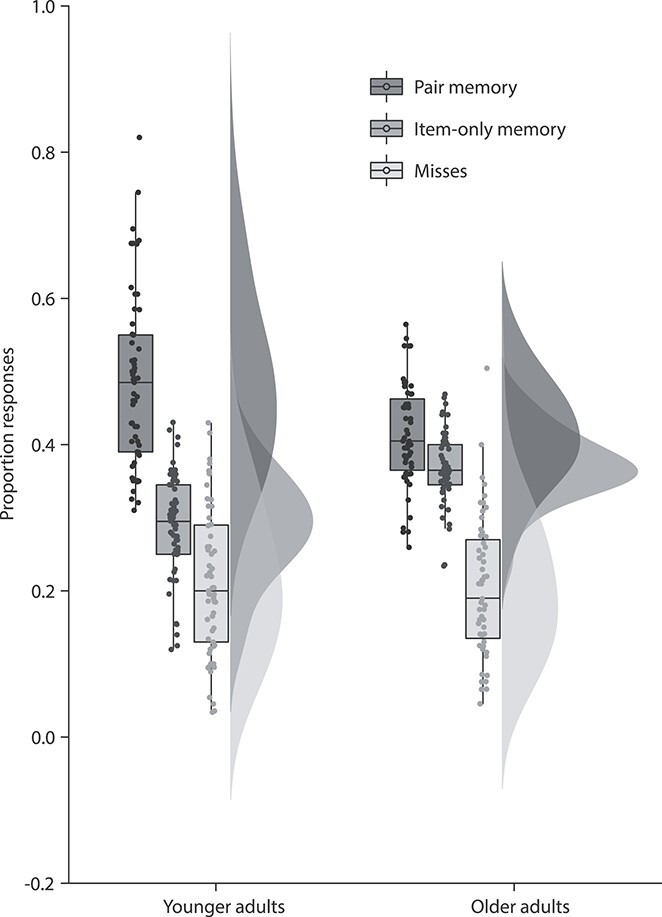
The proportion (*y*-axis) of misses (light gray), item–only (medium gray), and pair (dark gray) responses for each age group (*x*-axis). The plot illustrates comparable item memory performance across age groups, as indicated by age-invariant miss rates, and age-related impairments in pair memory, as reflected in higher item–only and lower pair response rates in the older adults. Box plots represent the interquartile range (first and third quantile), with dots representing individual participants. The horizontal bar indicates the median and half-violin plots illustrate the sample density.

To investigate age differences in memory performance, response rates were analyzed in a mixed-effects ANOVA with age (younger/older) as between- and memory level (miss/item–only/pair) as within-subject factors. A main effect of memory level (*F*(2, 220) = 143.6, *P* < 0.001), with response rates increasing from misses (*M* = 0.21, SD = 0.10), to item–only (*M* = 0.33, SD = 0.07), to pair memory (*M* = 0.45, SD = 0.11; see Fig. 2), was found. The low overall miss rate and the linear increase suggests that participants could indeed recognize the items and had some level of pair memory. Furthermore, an age-by-memory level interaction was found (*F*(2, 220) = 14.5, *P* < 0.001). Follow-up comparisons showed no difference in miss rate between age groups (*P* > 0.66), indicating comparable overall item recognition memory. However, there was a significant group difference in item–only response rates (*t*(100) = −6.94, *P* < 0.001), suggesting that older adults (*M* = 0.37, SD = 0.05) more often recognized the item without remembering the associated context compared to younger adults (*M* = 0.29, SD = 0.07). At the same time, pair response rates differed significantly (*t*(91.19) = 4.02, *P* < 0.001), indicating that younger adults (*M* = 0.49, SD = 0.12) remembered the associated context more often than the older adults did (*M* = 0.41, SD = 0.07). Thus, in line with the common observation in the literature, item memory did not differ between age groups. Instead, the age differences were evident in the proportion of correct and incorrect pair responses, with older adults clearly being impaired in retrieving the item–context association (Fig. 2). Since we did not assess memory for single contexts, we cannot completely rule out that part of the observed age differences reflect memory differences for the contextual information itself. However, given that each context was presented 5 times during encoding and represented in 4 retrieval trials (assigned to 2 match and 2 mismatch conditions at test), we contrasted the proportion of contexts for which the pair information was never correctly remembered on any of the retrieval trials. An independent samples *t-*test demonstrated that there were no differences between age groups (*P* < 0.14; younger adults: *M* = 0.10, SD = 0.10; older adults: *M* = 0.15, SD = 0.21), suggesting that older adults did not have inferior memory for the contexts per se relative to the younger adults.

### Theta and alpha/beta power support associative memory formation

**Fig. 3:**
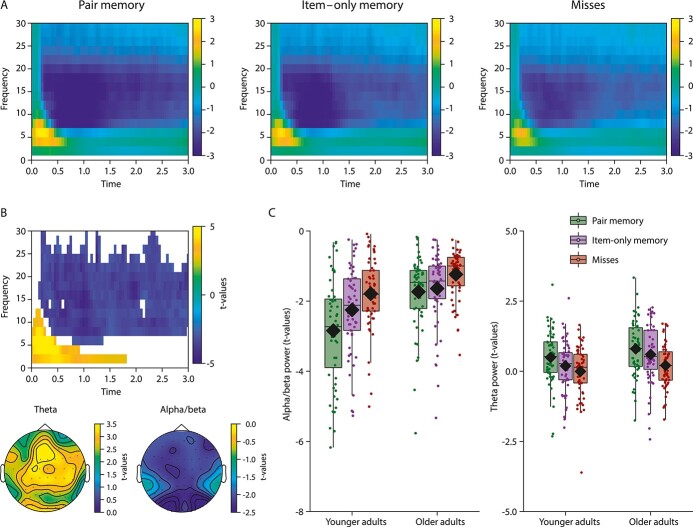
a) Time (*x*-axis)—frequency (*y*-axis) representations illustrating the theta and alpha/beta band responses during encoding for subsequently remembered object-scene associations (i.e. pair memory), for objects later remembered without memory for the associated scene (i.e. item–only memory), and for misses. Data were averaged over all channels. b) Illustration of the identified time (*x*-axis) and frequency (*y*-axis) cluster during encoding showing linear increases (relative to a prestimulus period) in theta power and decreases in alpha/beta power as a function of the amount of associative information available at retrieval (misses < item–only memory < pair memory). The topographical distributions of the effects are presented at the bottom. Data were averaged over time points for which the effects were statistically significant (theta: 0–1.8 s; alpha/beta: 0.2–3 s). c) For illustrative purposes only, we present the linear increase in alpha/beta desynchronization (left) and theta synchronization (right) across retrieval outcomes differing in the amount of associative information available. Data were averaged over trials within a frequency-and-region of interest defined according to the significant clusters. Box plots represent the interquartile range (first and third quantile), with dots representing individual participants. The rhombus indicates the mean and the horizontal bar indicates the median.

Time–frequency representations (Fig. 3a) of the encoding-related neural activity, averaged over all channels for illustrative purposes, demonstrate a power increase in the theta frequency range (~2–6 Hz) followed by a power decrease in the alpha/beta frequency range (~8–25 Hz). A similar oscillatory pattern is evident across all three conditions. To isolate differences in power during the formation of memories depending on subsequent retrieval outcome, we computed the change in power relative to a prestimulus period (−0.7 to −0.2 s) within participants and conducted linear regressions across the 3 conditions (i.e. misses, item–only memory, pair memory) on the group level. A positive (*P* < 0.001) and a negative (*P* < 0.001) cluster were identified, reflecting a reliable linear increase in theta synchronization and alpha/beta desynchronization as a function of the degree of associative information available at retrieval (misses < item – only memory < pair memory). Both effects showed widespread distributions, comprising most channels (Fig. 3b). The positive effect, spanning a narrower frequency range (~2–6 Hz) in the theta range, was evident early in the epoch (~0–2 s) and had a more fronto-central maximum (see Fig. 3b and c). The negative effect, spanning a broad frequency range (~8–25 Hz) in the alpha/beta range, was sustained over the full epoch (~0.2–3 s) with a posterior-occipital maximum. The two clusters were used as FROI for the following single-trial analyses.

### Single-trial theta and alpha/beta power predicts retrieval outcome in older and younger adults

**Fig. 4:**
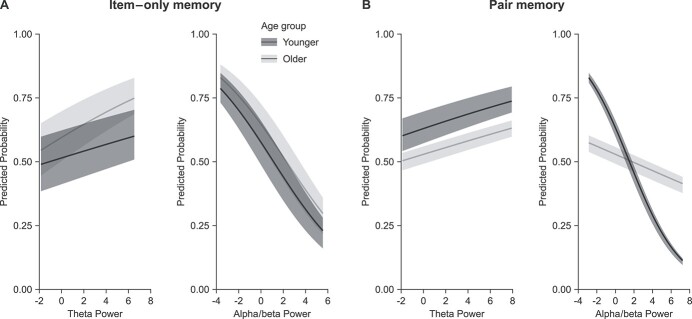
a) The effect of theta and alpha/beta power on the predicted probability for item–only memory (miss vs. item–only memory). Note that the theta effect as well as age groups are plotted for illustrative purposes only. Theta power did not reliably predict item–only memory in any age group. Alpha/beta power predicted item–only memory equally well in older and younger adults. b) The effect of theta and alpha/beta power on the predicted probability for pair memory (item–only memory vs. pair memory). For the theta effect, age group is included for illustrative purposes only. Theta power reliably predicted pair memory equally well in both age groups, whereas alpha/beta power predicted pair memory less well in the older than the younger adults.

To further understand the within-person relationship between the power modulations during memory formation and subsequent retrieval outcome, we used mixed-effects logistic regression to predict item–only and pair memory with theta and alpha/beta power on the single-trial level. To this end, we extracted baseline-normalized, single-trial power, averaged within the two FROIs and *z*-transformed across trials within each participant. The single-trial data were then entered into 2 separate models, 1 predicting item–only memory (i.e. miss vs. item–only memory; item–only model) and 1 predicting pair memory (i.e. item–only vs. pair memory; pair model). To assess age differences in the relationship between power modulations and retrieval outcome, age group was included as an additional predictor. Theta synchronization reliably predicted pair (*P* = 0.01) but not item–only (*P* = 0.09) memory. Alpha/beta desynchronization, on the other hand, reliably predicted both item–only (*P* < 0.001) and pair (*P* < 0.001) memory. In addition, while no other interaction terms were significant, an alpha/beta-by-age interaction (*P* < 0.001) was evident in the pair memory model (see Table 1 for parameter estimates). Figure 4 illustrates the effect of theta and alpha/beta power on the predicted probability of item–only memory and pair memory across age groups.

Furthermore, age group was a reliable predictor for both item–only (*P* = 0.005) and pair (*P* < 0.001) memory. However, the main effect of age in the item–only model parallels a significantly higher item–only response rate in the older adults (see “Older adults show reduced pair memory performance”). Thus, this observation is most likely a consequence of reduced pair memory rather than superior item recognition in older age.

Both the item–only (Log Likelihood = −7186.6, AIC = 14,387, BIC = 14,439, conditional *R*^2^ = 0.11) and the pair (Log Likelihood = −10,916, AIC = 21,846, BIC = 21,900, conditional *R*^2^ = 0.06) models significantly outperformed models without EEG variables as predictors (item–only: *X*^2^(4) = 208.36, *P* < 0.001; pair: *X*^2^(4) = 261.47, *P* < 0.001) and intercept-only models (item–only: *X*^2^(5) = 215.98, *P* < 0.001; pair: *X*^2^(5) = 300.17, *P* < 0.001).

## Discussion

In the present study, we investigated age differences in the oscillatory mechanisms of item–only as compared to pair memory formation. First, we replicated the common observation of age-invariant item memory in parallel with greater declines in associative memory in older compared to younger adults (Fig. 2; see also Karlsson et al. 2021, for the same pattern of results with corrected recognition scores in the same sample). Next, we showed that on the group level, theta synchronization and alpha/beta desynchronization during memory formation increased linearly with the amount of information available at retrieval (misses < item – only memory < pair memory; Fig. 3). We then used within-person fluctuations in theta and alpha/beta power between single trials to predict subsequent item–only and pair memory while assessing the effect of age.

Single trial theta power only predicted subsequent pair memory, but not item–only memory (Fig. 4). While we acknowledge that null findings must be interpreted with caution, in line with our expectations stronger theta synchronization during encoding increased the probability for retrieving the item–context association. These results agree with the notion that theta supports associative memory formation. However, contrary to the associative deficit hypothesis, we found no effect of age on the predictability of theta power for subsequent pair memory.

In contrast, alpha/beta power predicted both subsequent item–only memory (i.e. miss vs. item–only memory) and pair memory (i.e. item–only memory vs. pair memory; Fig. 4) in both age groups. Thus, in line with the observation on the group level, the probability for retrieving more information about an episode (misses < item – only memory, and item–only memory < pair memory) also increased with stronger alpha/beta desynchronization on a trial-by-trial basis. Thus, we extend recent work by showing that alpha/beta desynchronization during learning also scales with the amount of information that can be subsequently recovered for a given trial (cf. Karlsson et al. 2020; Martín-Buro et al. 2020). Finally, we observed that the predictability of single trial alpha/beta power for successful pair memory was reliably reduced in the older compared to the younger adults (Fig. 4; see also Karlsson et al. 2020). Thus, attenuated alpha/beta desynchronization in older adults during memory formation, presumably reflecting a reduction in the depth of information processing, contributes to age-related impairments in episodic memory.

### Theta synchronization supports associative memory formation

The synchronization of theta-band activity has been implicated in the formation and retrieval of memories relying on the associative binding of distinct elements of an event (see Clouter et al. 2017; Herweg et al. 2020 for reviews). In addition, encoding-related theta power modulations associated with subsequent memory performance have been demonstrated in the hippocampus, in intracranial EEG recordings (Lega et al. 2012; Lin et al. 2017) and via source localization of scalp-recorded EEG (Hanslmayr et al. 2011). In addition, successful encoding of associations has been related to increased theta phase coherence between frontal and posterior channels (Summerfield and Mangels 2005) and between the prefrontal cortex (PFC) and the hippocampus (Backus et al. 2016). Thus, the theta rhythm supports memory processing within and between regions functionally and computationally involved in episodic memory (for a review, see Fell and Axmacher 2011). Accordingly, we observed that theta synchronization during encoding was predictive of subsequent retrieval of associative information. Importantly, single-trial theta power predicted whether the item would later be retrieved with or without memory for its associated context. At the same time, theta power did not reliably predict whether the item itself would be remembered or not. The linear increase in theta power observed on the group level may then be driven by the power difference between item–only and pair memory, which may become elevated when averaging over trials. The logistic mixed model, however, considers within-person single-trial power fluctuations. Thus, our findings suggest that theta synchronization is a key mechanism supporting item–context binding, adding to our understanding of the role of theta in episodic memory formation. Given the limitation of interpreting the nonsignificant effect of theta on item–only memory, future work may focus on directly contrasting item and pair memory formation using a paradigm where the 2 can be more clearly separated.

Since we did not localize the source of the theta effect, and it is unclear whether scalp-recorded theta originate from hippocampal (Ekström et al. 2005) or cortical (Raghavachari et al. 2006) generators, it is difficult to infer the exact mechanistic role of the present theta effect. Although the neural source is impossible to derive from the topography of scalp EEG, the fronto-central maximum of the effect may suggest that we are observing long-range communications between the MTL and prefrontal and/or posterior regions, reflecting top-down organizational control over memory formation (for review, see Klimesch 1999; Fell and Axmacher 2011). However, theta SMEs have been related to the MTL (Hanslmayr), and hippocampal projections may drive cortical theta (Ekström et al. 2005) and bias activity in cortical neural assemblies (Sirota et al. 2008). Thus, the effect may also mirror mechanisms more directly involved in hippocampus-dependent associative binding.

### Alpha/beta desynchronization indexes the depth of information processing

Recent theoretical accounts hold that alpha/beta desynchronization reflects a general mechanism promoting efficient information processing (Hanslmayr et al. 2012; Hanslmayr et al. 2016; see also Klimesch et al. 2007). High synchrony in neural firing across task-relevant networks induces a low signal-to-noise ratio and masks the signal of interest (Averbeck et al. 2006; Mitchell et al. 2009; Churchland et al. 2010). Hence, for the system to efficiently convey the message within and between task-relevant neural assemblies, desynchronization of these neural circuits is necessary to promote the flow of information. Alpha/beta desynchronization is proposed to reflect this reduction in synchronized activity between task-relevant neural assemblies, promoting deep processing of information necessary for the construction of highly detailed memories (Hanslmayr et al. 2012).

Accumulating evidence has demonstrated that alpha/beta desynchronization during encoding and retrieval increases linearly with the amount of information available, operationalized either in terms of memory performance (Karlsson et al. 2020; Martín-Buro et al. 2020) or in terms of the specificity of (re)activated neural representations (Griffiths et al. 2019). In addition, alpha/beta desynchronization has been associated with the depth of elaborative encoding (Hanslmayr et al. 2009), and it was recently shown that the magnitude of alpha/beta power decreases during encoding predicted the magnitude of alpha/beta power decreases at retrieval (Griffiths et al. 2021). However, thus far, whether alpha/beta desynchronization during encoding predicts subsequent item–only versus pair memory in a linear fashion has not been directly tested (cf. Karlsson et al. 2020; Martín-Buro et al. 2020). Thus, our study adds a missing piece to the puzzle by demonstrating that alpha/beta desynchronization during memory formation predicts how much information can later be recovered at retrieval. Although we cannot assess alpha/beta desynchronization at retrieval in the present data, we can infer the amount of information recovered based on memory performance at test. Thus, in line with previous work (Hanslmayr et al. 2012; Griffiths et al. 2019), we propose that alpha/beta desynchronization reflects the depth of information processing during the initial experience of an event, which determines the contents of the stored memory trace and ultimately how much information can later be recovered about the initial event (Tulving and Thomson 1973).

### Attenuated alpha/beta desynchronization during memory formation contributes to impaired associative memory in older age

The main goal of this study was to investigate the neural mechanisms proposed to underlie/contribute to the processes central to 2 long-standing hypotheses accounting for age-related declines in episodic memory, namely associative binding and the depth of information processing (Naveh-Benjamin 2000; Craik and Rose 2012). In particular, we first assessed whether associative memory decline in older age is linked to a binding deficit during encoding, as reflected in attenuated theta synchronization. Second, we examined whether reductions in the depth of information processing, as indexed by alpha/beta desynchronization, contributes to age-related declines in the amount of information that can be recovered at retrieval.

Theta power did not predict item–only memory in any age group, but predicted pair memory equally well in older and younger adults. Thus, theta synchronization contributed to associative memory formation in particular, but nonetheless could not account for reduced associative memory in the older adults. Our findings are in line with Sander et al. (2020) who also did not observe age differences in theta SME, therefore not supporting the hypothesis that altered theta synchronization underlies age-related impairments in associative binding during memory formation. However, as elaborated above, it is possible that the observed theta effect mirrors long-range interactions between the hippocampus and the PFC, reflecting top-down control of memory organization rather than hippocampally mediated associative binding (see Sander et al. 2020 for similar topography findings). The fronto-central maximum of the effect may support this speculation (Nyhus and Curran 2010). Another likely possibility is that theta synchronization is necessary but not sufficient for associative binding. Accumulating evidence suggests that the precise interaction between theta and high-frequency gamma (<30 Hz) band activity represents a neural mechanism reflecting the formation of associations (e.g. Lisman and Jensen 2013; Staudigl and Hanslmayr 2013; Heusser et al. 2016; Köster et al. 2019). In particular, impairments in associative binding in older age may rather be expressed as time-shifted cross-frequency coupling of gamma power to the phase of the theta rhythm than as reductions in theta SME. In fact, in this same sample we have recently shown that the precise coupling of cortical gamma power to a specific theta phase optimal for successful pair memory formation was compromised in the older adults and predicted subsequent pair memory performance. At the same time, there were no age differences in the overall presence of rhythmic theta activity (Karlsson et al. 2021). In concert, these observations strongly suggest that the recruitment of theta activity per se does not drive age-related impairments in associative memory formation, but rather the precise interaction between the theta rhythm and activity in higher frequency bands.

In contrast, alpha/beta power modulations during encoding predicted item–only memory in both age groups. In parallel, alpha/beta power differentiated between trials for which only the item and trials for which the item–context association was later remembered less well in the older relative to the younger adults. These observations are in line with Sander et al. (2020), who reported a link between reduced IFG volume, attenuated alpha/beta desynchronization, and reduced associative memory. Taken together, our present findings strongly indicate that reductions in the depth of information processing, as reflected in attenuated alpha/beta desynchronization, contribute to reduced associative memory performance in older age. Apparently, older adults are generally able to process item information deeply enough to successfully recognize the item at test. However, they are impaired when deep processing of both item and context is necessary to construct highly detailed episodic memories. Thus, we demonstrate age differences in a neural mechanism proposed to reflect the depth of information processing during encoding and show that age differences in both our behavioral and neural measures scale with the difficulty of the task (i.e. item recognition < pair memory). In addition, it is noteworthy that these age differences were evident despite the participants being provided with an encoding strategy aiming to aid elaborative encoding. Thus, in line with the levels-of-processing framework (Craik and Lockhart 1972; Craik and Rose 2012), reduced information processing capacity in older age impairs elaborative encoding and contributes to impaired episodic memory in older age.

Of note, the discrepancies reported across this and previous studies (cf. Strunk and Duarte 2019; Karlsson et al. 2020; Sander et al. 2020) are presumably due to differences in task design. Sander and colleagues used repeated cued recall with feedback, explicitly attempting to reduce age differences during learning, which may have made elaborative encoding easier over learning rounds, as reflected in stronger alpha/beta desynchronization. Our results, on the other hand, may instead reflect age differences in the “one-shot” encoding of a specific event into long-term memory. Furthermore, Strunk and Duarte (2019) used an item recognition task with confidence ratings to delineate familiarity and recollection at retrieval. However, especially given age differences in such metacognitive measures (e.g. Shing et al. 2009; Fandakova et al. 2013), inferring recollection based on confidence ratings may not account for the contribution of familiarity. Here, we explicitly assessed both item recognition and associative memory, which allowed us to better isolate memories depending on recollection, and we provide strong evidence for age differences in alpha/beta desynchronization during associative memory formation. In concert with other studies directly contrasting item and pair memory encoding in older and younger adults (e.g. Dennis et al. 2008; Saverino et al. 2016), this indicates that the way age differences appear depends on whether the task relies on associative memory and the extent to which recollective processes can be isolated.

Finally, reduced elaborative encoding and attenuated alpha/beta desynchronization in older adults also conform to the proposal that the representation of information is less specific in older compared to younger adults due to reductions in neural resources and an overall noisier system in older age (Li et al. 2000, 2006; see also Li et al. 2005). Interestingly, converging evidence suggests that the way information is represented neurally becomes less specific and less predictive of memory outcomes in older adults (e.g. Saverino et al. 2016; Morcom and Henson 2018; Koen et al. 2020). At the same time, alpha/beta desynchronization promotes representational specificity (Griffiths et al. 2019) and, as we have recently shown, attenuated alpha/beta desynchronization at retrieval is related to the recovery of less specific memories in older compared to younger adults (Karlsson et al. 2020). Here we show that attenuated alpha/beta desynchronization at encoding contributes to age differences in associative memory by determining the amount of information available at retrieval. Thus, our observations bridge previous work by showing that a key mechanism, that globally regulates efficient information processing, is compromised in older age and has downstream effects on the quality of the recovered memory trace (see Sander et al. 2021 for a review).

## Conclusion

To conclude, we show that alpha/beta desynchronization and theta synchronization during memory formation differentially shape the content and quality of memory traces. Alpha/beta desynchronization supported successful memory formation independent of item or pair retrieval success, thus reflecting a general mechanism representing the depth of information processing. Modulations in theta synchronization on the other hand, predicted subsequent pair memory but not item–only memory, in line with its specific role in the formation of associative memories. Behaviorally, age groups differed in associative memory performance, but not in item recognition. However, on the neural level, age differences were only found in alpha/beta desynchronization, which predicted pair memory less well in older than in younger adults. Thus, we propose that less deep elaboration of incoming information, as reflected in attenuated alpha/beta desynchronization, results in the construction of less detailed memories and determines the amount of information that can be recovered at retrieval.

## Data Availability

Code and data will be made publicly available upon publication.
